# Frequent gain of the human telomerase gene *TERC* at 3q26 in cervical adenocarcinomas

**DOI:** 10.1038/sj.bjc.6603253

**Published:** 2006-07-18

**Authors:** S Andersson, K-L Wallin, A-C Hellström, L E Morrison, A Hjerpe, G Auer, T Ried, C Larsson, K Heselmeyer-Haddad

**Affiliations:** 1 Department for Clinical Science, Intervention and Technology, Division of Obstetrics and Gynecology, Karolinska University Hospital-Huddinge, Karolinska Institutet, Stockholm SE-141 86, Sweden; 2 Department of Molecular Medicine and Surgery, Karolinska Institutet, Karolinska University Hospital-Solna, CMM L8:0, Stockholm SE-171 76, Sweden; 3 Department of Gynecologic Oncology, Radiumhemmet, Karolinska University Hospital-Solna, Stockholm SE-171 76, Sweden; 4 Abbott Molecular, 1300 East Touhy Avenue, Des Plaines, IL 60018, USA; 5 Department of Oncology-Pathology, Karolinska University Hospital Solna, CCK, Stockholm SE-171 76, Sweden; 6 Genetics Branch, Center for Cancer Research, National Cancer Institute/NIH, Building 50, Room 1408, 50 South Drive, Bethesda, MD 20892-8010, USA

**Keywords:** cervical adenocarcinoma, human papillomavirus, interphase fluorescence *in situ* hybridisation, chromosome arm 3q, telomerase, early detection

## Abstract

The level of genomic amplification of the human telomerase gene *TERC*, which maps to chromosome band 3q26, was determined in primary cervical adenocarcinomas. Interphase nuclei prepared from archival material of 12 primary cervical adenocarcinomas, eight of which were human papillomavirus positive, were hybridised with a triple colour probe set specific for centromeres of chromosomes 3 and 7 and the *TERC* gene. We observed high proportions of nuclei with increased absolute copy numbers for *TERC* in all tumours (mean 3.3; range 2.3–5.2). Amplification of the human telomerase gene *TERC* is a consistent aberration in cervical adenocarcinomas. Therefore, application of our probe set may provide an objective genetic test for the assessment of glandular cells in Pap smears and hence for the diagnosis of cervical adenocarcinomas.

Cervical cancer is a disease that mainly affects women in the forth or fifth decade of life. According to the International Agency for Research on Cancer (IARC), it is the second most common cancer in women worldwide, and in developing nations it is the most common cancer in women (Parkin *et al*, 2001). Two main types of cervical cancer are described, that is, squamous cell carcinoma and adenocarcinoma. Cervical adenocarcinomas comprise 20% of all cervical cancers that are diagnosed (Vizcaino *et al*, 1998). Importantly, in the last few decades the incidence of cervical adenocarcinoma among young women has been steadily increasing (Vizcaino *et al*, 1998; Bergström *et al*, 1999; Smith *et al*, 2000; Sasieni and Adams, 2001); however, the reasons for this increase are presently unknown. Applications of clinical screening programmes that have led to a decrease in squamous cell carcinoma have only shown a limited effect on the occurrence of adenocarcinomas. This is owing to the fact that the sensitivity for detecting precursor lesions of adenocarcinomas is much lower than for the detection of precursor cells of squamous carcinomas. This can be partly explained by the difficulty in the morphological assessment of highly differentiated glandular cells, which can be derived from adenocarcinomas of the endocervix. In addition to these difficulties, the endocervix is less accessible for cytological sampling (Bergström *et al*, 1999).

Epidemiological and molecular biological studies have shown that infection with high-risk human papillomavirus (HPV) is an important aetiological agent in the pathogenesis of cervical adenocarcinoma (Bosch *et al*, 1995; Bosch and Munoz, 2002). More than 99% of squamous cell carcinomas harbour oncogenic types of HPV, with HPV 16 being the most frequently encountered (Walboomers *et al*, 1999). Partly in contrast, we and others have found HPV DNA in 30–90% of cervical adenocarcinomas, with HPV 18 as the predominant type (Tenti *et al*, 1996; Andersson *et al*, 2003a, 2003b; Clifford *et al*, 2003; Zielinski *et al*, 2004; Castellsague *et al*, 2006).

Although tightly linked to cervical cancer development, HPV infection alone is not sufficient for oncogenic transformation, and other factors than HPV are required for the malignant transformation towards cervical adenocarcinomas (Skyldberg *et al*, 1999; Pirog *et al*, 2000). The application of cytogenetic and molecular cytogenetic techniques to study genomic alterations have identified frequent gains involving the long arm of chromosome 3 (Yang *et al*, 2001). This observation is in concordance with previous findings of recurrent 3q amplification in squamous cell carcinomas (Heselmeyer *et al*, 1996, 1997a). The value of detection of genomic amplification of 3q as a biomarker for progression during uterine tumorigenesis was therefore evaluated in several studies. The results showed that amplification of *TERC* is almost invariably found in Pap smears assessed as HSIL (i.e., high squamous intraepithelial lesions). In addition, a more recent study unambiguously established the central role of 3q for progression from low-grade dysplastic lesions to higher grades and to invasive carcinomas and showed that the gain of 3q can occur in morphologically normal Pap smears of women who developed cervical carcinomas after only a short latency (Heselmeyer-Haddad *et al*, 2003, 2005). Gain of chromosomal region 3q is also recurrently observed in other types of cancer, including tonsillar cancer and carcinomas of the anogenital region that are both associated with HPV DNA (Heselmeyer *et al*, 1997b; Dahlgren *et al*, 2003; Stoltzfus *et al*, 2005).

In this study, we have determined *TERC* copy number gain in cervical adenocarcinomas using interphase fluorescence *in situ* hybridisation (FISH) on cytospins prepared from single-cell suspensions of disintegrated archival paraffin-embedded tissue and correlated the findings to the presence of HPV infection and the clinical course.

## PATIENTS AND METHODS

### Tumour material

The study includes formalin-fixed paraffin-embedded tumour tissue specimens from 12 primary cervical adenocarcinomas diagnosed and surgically treated at the Karolinska University Hospital-Huddinge during 1992–2000 (Table 1). All tumour cases were identified from the Swedish Central Cancer Registry organised by the National Board of Health and Welfare. This registry includes all cases of malignant tumours diagnosed histopathologically after 1959, whereby each tumour is identified by a topographical and histopathological code. All tumour samples were collected with informed consent and approval from the local ethics committee.

The clinical information for each case is detailed in Table 1 and has been published previously (Andersson *et al*, 2003a). The histopathological diagnosis was based on the WHO criteria. The 12 tumours were classified as cervical adenocarcinomas and all were without any squamous cell component. Furthermore, the four HPV-negative tumours (T4, T5, T8, T9) were histopathologically re-evaluated and revealed typical morphology of cervical adenocarcinoma in agreement with the initial diagnosis. Immunostainings with vimentin and carcinoembryonic antigen (CEA) were carried out. Cases T4, T5 and T8 were strongly positive for CEA and negative for vimentin, in agreement with the common pattern for adenocarcinoma of cervical origin as reported by McCluggage *et al* (2002). Case T9 showed atypical staining pattern (CEA was negative and vimentin positive); however, the morphology was strongly suggestive of cervical origin as judged by several independent histopathologists. Seven tumours were well differentiated (58%), one was moderately differentiated (8%) and four tumours were poorly differentiated (33%). Clinical staging was according to the International Federation of Gynecology and Obstetrics (FIGO) classification for cervical cancer (Benedet *et al*, 2000). At initial diagnosis, 10 tumours were classified as stage I (83%) and two tumours as stage II (17%). Two patients initially exhibited lymph node involvement (17%). All patients were previously sampled for Pap smear analysis. All but one Pap smear (which was evaluated as showing signs of cellular inflammation) were assessed as normal at the time of the most recent cytological evaluation (Table 1). Re-evaluation of the Pap smears confirmed the initial diagnoses in all cases (Andersson *et al*, 2003a). All patients were retrospectively followed up from the time of diagnosis until June 2005, and disease recurrence and survival data recorded.

### HPV status

Results from HPV screening analyses have been previously published for all cases (Andersson *et al*, 2003a). Briefly, the analyses were performed on extracted DNA obtained from sections of paraffin blocks, of which the preceding section had been used for morphological diagnosis. A fragment of 139–148 bp was amplified from the L1 region with GP5+/GP6+ primers, HPV typed by direct DNA sequencing and comparison to known HPV sequence databases using the BLAST algorithm (www.ncbi.nlm.nih.gov/BLAST). Eight of the 12 tumours were thus found to be HPV-positive; five tumours were infected with HPV 18, two with HPV 16 and one with HPV 45 (Table 1). In the remaining four tumours, HPV was not detected.

### Preparation of nuclei suspensions for FISH analysis

Single-layer nuclei preparations for interphase FISH hybridisations were prepared using the Hedley method with modifications (Castro *et al*, 1993). A 50-*μ*m section was cut from each of the 12 formalin-fixed paraffin-embedded tumour tissue samples. After deparaffinisation in xylene, the section was rehydrated in an ethanol series and in distilled water, and the section was disintegrated in 500 *μ*l of 0.1% protease/1 × PBS (Protease : Type XXIV, Bacterial, P 8038, Sigma, St Louis, MO, USA and Dulbecco's 1 × PBS, Life Technologies, Rockville, MD, USA) at 45°C for 45–60 min. The reaction was stopped by adding 500 *μ*l 1 × PBS at room temperature. The sample was then filtered through a nylon membrane (CN 051, DAKO, Glostrup, Denmark), centrifuged and resuspended in 1 × PBS. Cytospin slides were prepared using a Shandon Cytospin® centrifuge and fixed in an ethanol series.

### FISH

Triple-colour FISH analysis was performed on each case using the following probe set: a centromere-specific probe for chromosome 7 (CEP®7); a centromere-specific probe for chromosome 3 (CEP3) and a contig consisting of four overlapping BAC clones containing the *TERC* gene at chromosomal location 3q26. All probes were obtained from Vysis/Abbott Molecular Inc. (Des Plaines, IL, USA). The details for this probe set, its sensitivity and specificity, as well as experimental conditions were published previously (Heselmeyer-Haddad *et al*, 2003). In short, CEP7 was labelled with Spectrum Aqua™ (SA), CEP3 with Spectrum Green™ (SG) and the *TERC* contig with Spectrum Orange™ (SO), using chemical labelling as described (Bittner *et al*, 1996). Before hybridisation, the cytospin slides were pretreated with a pepsin digestion and fixed in an ethanol series. Slides were denatured in 70% formamide/2 × SSC for 3.5 min at 80°C. The probes were denatured according to the manufacturer's recommendations. After overnight hybridisation at 37°C, the slides were first washed four times in 50% formamide/2 × SSC at 45°C (once for 3 min and three times for 7 min), followed by washes in 2 × SSC at 45°C for 5 min and in 2 × SSC/0.1% NP40 at 45°C for 5 min. The slides were counterstained with 4,6-diamidino-2-phenylindole (DAPI), and subsequently embedded in an antifade solution.

### Scoring of FISH results

Fluorescence *in situ* hybridisation and image analyses were carried out using a Leica DM-RXA fluorescence microscope (Leica, Wetzlar, Germany) equipped with custom optical filters for DAPI, SA, SG and SO (Chroma Technologies, Brattleboro, VT, USA) and × 40 Plan Apo (NA 1.25) objective. Images were taken in areas of optimal cell density with minimal cellular clumps using an ORCA ER (IEEE1394 I/F) digital camera (Hamamatsu, Bridgewater, NJ, USA). Leica Q-Fluoro was used to acquire multifocus images for each of the DAPI, SA, SG and SO optical filters. Ten to 16 images were acquired and signal enumeration was performed on these digital images for 208–641 nuclei for each case. The counted signals were listed and evaluated in Excel-based customised software.

Nuclei that could not be evaluated (e.g., because of insufficient hybridisation or overlapping nuclei) were excluded from further analysis. The results for all ‘countable’ nuclei were registered in relocation charts in the form of patterns for the entire probe panel. For example, the pattern (2-3-3) refers to two signals for CEP7, three signals for CEP3 and three signals for *TERC* in a given nucleus. Nuclei with normal signal numbers for the three probes (i.e., 2-2-2) were recorded as ‘diploid’, and nuclei with four signals for each probe (pattern 4-4-4) were considered ‘tetraploid’. The background level for the CEP7–CEP3–*TERC* probe panel was previously evaluated on cytological slides that contained nuclei from normal cervical cells (Heselmeyer-Haddad *et al*, 2003). This confirmed that deviation from the expected (2-2-2) pattern was seen in less than 2% of normal cells.

## RESULTS

### Frequent gain of *TERC* in cervical adenocarcinomas

**Figure 1:**
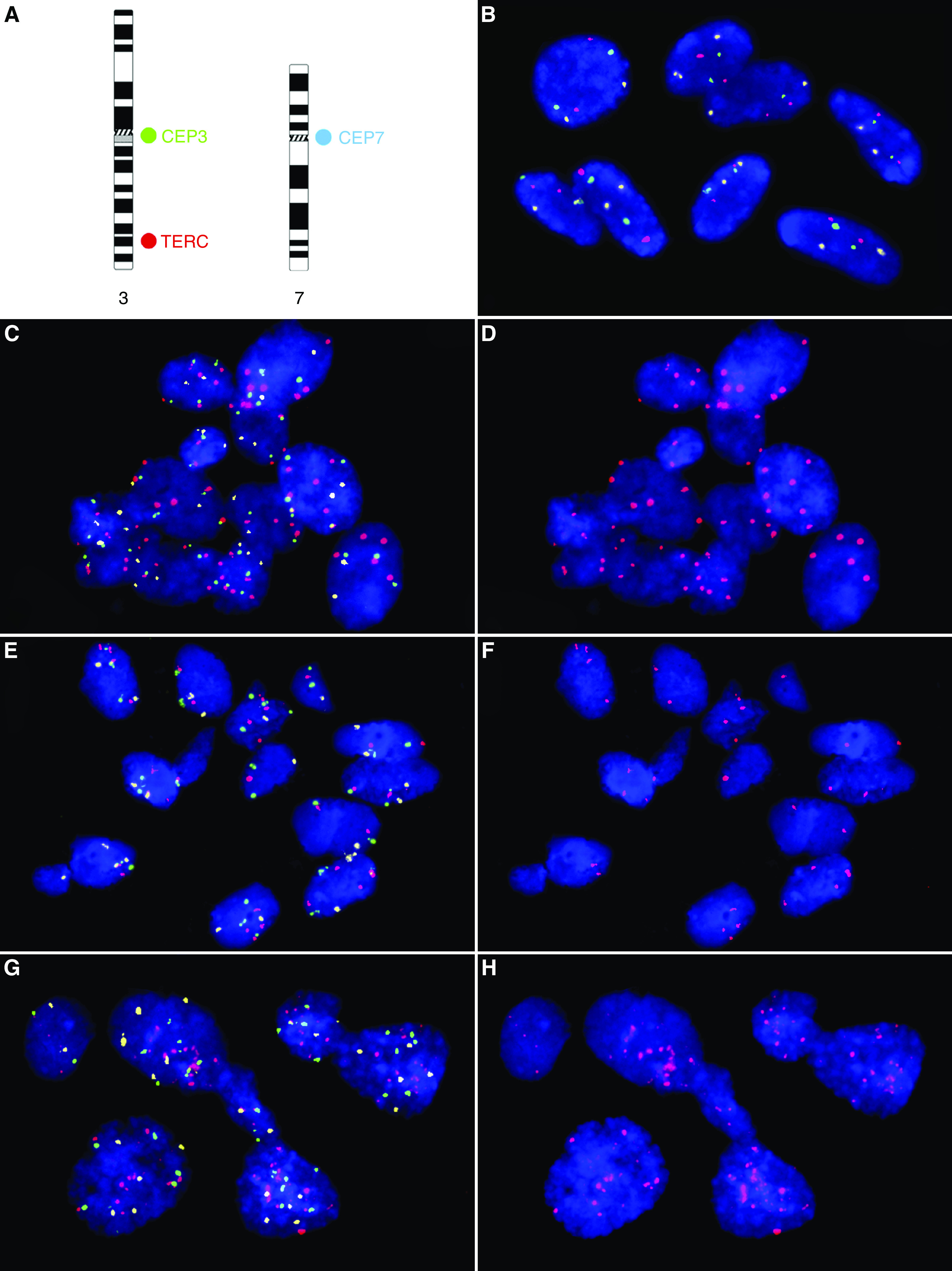
Interphase FISH analysis showing gain of *TERC* in 3q26 in cervical adenocarcinomas. (**A**) Chromosomal location of the triple-colour probe set: CEP 7 (labelled with Spectrum Aqua, which is pseudocoloured in yellowish-white in panels B, C, E and G), CEP 3 (Spectrum Green, pseudocoloured in green in panels B, C, E and G), and *TERC* (Spectrum Orange, pseudocoloured in red). (**B**) Normal nuclei with the expected pattern of two signals per probe per nucleus (2-2-2). (**C** and **D**) HPV-negative case number 4, (**E** and **F**) HPV-infected case number 6 and (**G** and **H**) HPV-positive case number 10. All cases exhibit varying extents of gain of *TERC*. The cell clusters in **C**, **E** and **G** and in **D**, **F** and **H** show identical cells with either the triple-colour probe set shown or with the *TERC* probe only, respectively.

**Figure 2:**
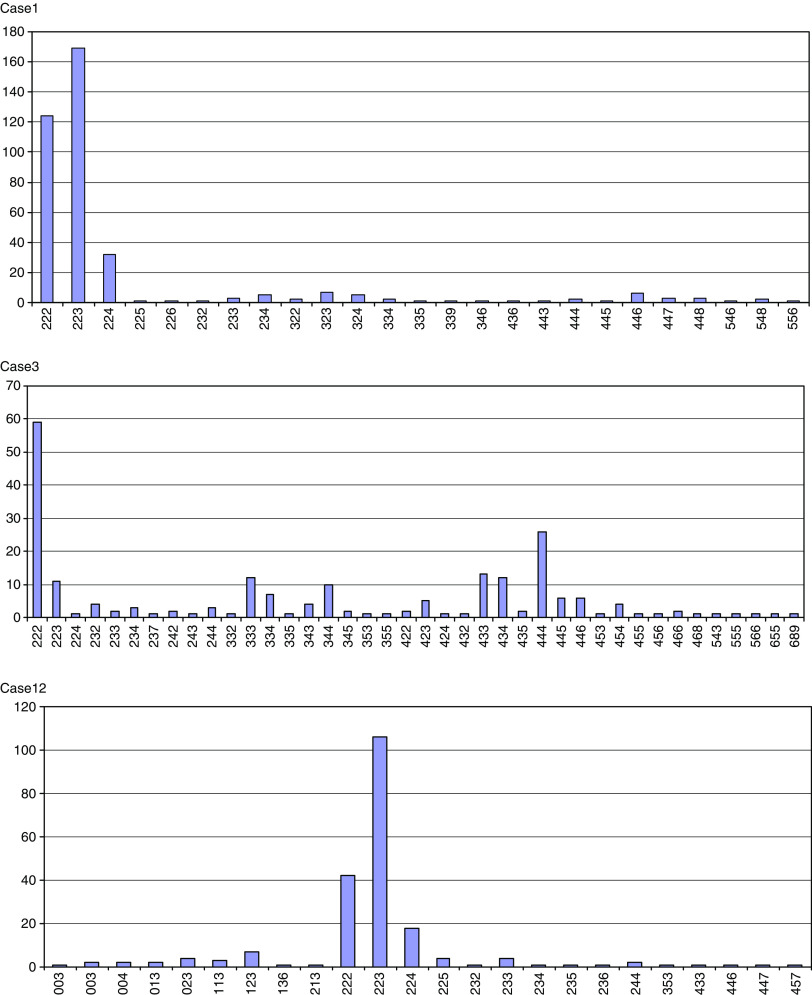
Diagrams illustrating the scoring results from interphase FISH analyses in cases 1, 3 and 12. Scoring patterns for CEP3–CEP7–*TERC* are given along the *x* axis and the numbers of detected nuclei with a given pattern are given on the *y* axis.

Twelve cervical adenocarcinomas (Table 1) were evaluated for copy number changes of the *TERC* locus at chromosomal band 3q26 using interphase FISH. The three-colour probe panel consisting of CEP7-CEP3-*TERC* was simultaneously hybridised to cytospin slides with interphase nuclei prepared from formalin-fixed tissue sections. All cases were successfully hybridised and analysed, whereby 208–641 nuclei per case were scored. Figure 1 shows representative hybridisations. Both normal ‘diploid’ patterns (2-2-2) and nuclei with nondiploid patterns were observed. The results are summarised in Table 2. In all 12 tumours, a significant proportion of ‘nondiploid’ nuclei was detected, and nine of the tumours exhibited >50% ‘nondiploid’ nuclei. In the individual tumours, nuclei with >2 *TERC* signals were found in similar frequencies as cells with ‘nondiploid’ pattern, and almost all nuclei with ‘nondiploid’ pattern exhibited >2 *TERC* signals. In the ‘nondiploid’ nuclei, a ‘tetraploid’ pattern (4-4-4) was not commonly observed. In 10 of the tumours, less than 1% of the nuclei exhibited (4-4-4) and in two cases (4-4-4) was seen in 4 and 12%, respectively (Table 2; exemplified for case 3 in Figure 2). Thus, gain of *TERC* was present in all tumours studied and was not an effect of ‘tetraploid’ status.

### Intratumour heterogeneity for gain and amplification of the *TERC* locus

We observed different FISH patterns in the individual cases. For several of the tumours, one or a few predominating patterns were seen (exemplified for cases 1, 3 and 12 in Figure 2). In addition, all cases exhibited varying proportions of different but recurrent ‘nondiploid’ patterns. Comparison of signal numbers recorded for *TERC vs* CEP7 and CEP3 showed increased relative *TERC* copies in the 12 cases studied (range 1.04–2.0; Table 2). The absolute mean number of *TERC* signals per nuclei ranged between 2.3 (case 11) and 5.2 (case 10), and in five of the tumours nuclei with more than 10 signals were repeatedly encountered (Table 2). Figure 1 illustrates nuclei of case 10 with amplifications of up to 20 *TERC* signals and more, together with 5–6 signals for CEP3 and CEP7 (Figure 1G and H), which shows that *TERC* can be highly amplified in certain cases. However, the more common pattern observed in most cases are low-level copy numbers of *TERC*, often on a still diploid background with patterns that can be reconciled with extra chromosomes of 3 or an isochromosome 3q formation.

### Gain of *TERC* and HPV infection

The clinical and follow-up information as well as HPV status of the 12 cervical adenocarcinomas are provided in Table 1. Eight cases were HPV-positive and in the other four HPV was negative. Very high proportions of ‘nondiploid’ nuclei with >2 *TERC* signals were demonstrated in HPV-positive (83–99%) as well as HPV-negative (86–99%) tumours. The absolute numbers of *TERC* signals per nucleus were similarly increased in HPV-positive (mean 3.4; range 2–22) and HPV-negative (mean 3.3; range 2–12) tumours. No apparent associations between *TERC* copy numbers and clinical characteristics, such as age at diagnosis, stage, differentiation, lymph node involvement or outcome at follow-up, were noted. Thus, gain of *TERC* is characteristic of cervical adenocarcinomas *per se*.

## DISCUSSION

In our study, we analysed a series of 12 primary cervical adenocarcinomas for gain or amplification of the human telomerase gene *TERC*, which maps to chromosome band 3q26. Eight of the 12 carcinomas (67%) were HPV-positive (five tumours were positive for HPV 18, two for HPV 16 and one for HPV 45) (Skyldberg *et al*, 1999; Andersson *et al*, 2003a). We then used FISH with a custom-designed probe panel that includes the human telomerase gene (*TERC*) to assess the potential of this genetic marker to ameliorate the morphological diagnosis of cervical adenocarcinomas. This is the first study that reports the consistent gain of the human telomerase gene *TERC* in cervical adenocarcinomas.

Yang *et al* (2001) analysed 20 cases of cervical adenocarcinomas with CGH and found that genetic alterations were evident in most of the cervical adenocarcinomas, including the gain of 3q, which was detected in 70% of these 20 tumours. From the findings of similar 3q amplifications in adenocarcinomas as reported for squamous cell carcinoma, it was proposed that similar mechanisms are involved in the development of the two types of cervical cancer (Yang *et al*, 2001). In our study, using FISH we detected a gain of 3q in all our cases. This difference is likely attributable to the different sensitivity of the respective techniques. Detection of a trisomy by CGH requires this numerical aberration to be present in at least 40% of the cells. Fluorescence *in situ* hybridisation, of course, detects changes on a single-cell basis and is therefore not sensitive to dilution. Additionally, small regional low-copy number amplicons might escape detection by CGH.

In our study, HPV infection was not detected in four of the cases, which could either reflect true HPV negativity or represent ‘false negatives’ owing to technical difficulties in HPV genome detection. In the current literature, HPV infection has not been reported in the same proportion in adenocarcinomas as in squamous cervical carcinomas (Tenti *et al*, 1996; Skyldberg *et al*, 1999; Andersson *et al*, 2003b; Clifford *et al*, 2003; Zielinski *et al*, 2004; Castellsague *et al*, 2006). Perhaps, oncogenic factors other than HPV are more likely to play a role in the malignant transformation of cervical adenocarcinomas (Skyldberg *et al*, 1999; Pirog *et al*, 2000).

The application of screening programmes has reduced the incidence of squamous carcinoma in Sweden substantially during the last decades (Mählck *et al*, 1994; Ponten *et al*, 1995). Yet, several studies suggest that a single Pap smear has a high false-negative rate (Macgregor *et al*, 1994). A review of evidence-based data revealed that as many as 50% of precancerous cervical lesions may be missed by a single Pap test (Sherman *et al*, 1994). Kinney *et al* (1998) showed that only 30% of women with histopathologically confirmed high-grade disease had corresponding atypia in their Pap smears. Against this background, a new screening strategy for cervical cancer has been presented in which HPV testing is combined with cytological examinations (Walboomers *et al*, 1999). However, screening methods currently utilised to detect preinvasive and early invasive squamous cervical cancer had only a limited effect in detecting precursor lesions of cervical adenocarcinoma.

Hildesheim *et al* (1999) classified women as having rapid-onset cervical cancer with the following criteria: a detection of cervical cancer within 5 years of at least one normal Pap smear or within 10 years after the first sexual intercourse. Our data showing that 10 of the 12 women had a normal Pap smear within 3 years before the diagnosis of invasive disease may support the hypothesis of a rapid-onset carcinoma. On the other hand, as the sensitivity of a Pap smear for the detection of cervical adenocarcinomas is very low (Krane *et al*, 2001), the rapid-onset hypothesis may not apply to our cases.

Cervical squamous cell carcinomas are defined by a nonrandom and recurrent distribution of genomic imbalances. In addition to HPV, the sequential transformation of cervical squamous epithelium requires the acquisition of additional copies of chromosome arm 3q. In the current study, we wanted to analyse and understand whether the gain of 3q plays a similar role in the tumorigenesis of cervical adenocarcinomas. Using a genomic probe for the *TERC* gene on chromosome band 3q26 in combination with two centromere-specific probes (CEP3 and CEP7), we showed high copy numbers of this locus in cervical adenocarcinomas. Gain or amplification of 3q was found in all cervical adenocarcinomas investigated. Therefore, the application of this probe set may provide an objective genetic test for the diagnosis of cervical adenocarcinomas and might assist in the interpretation of smears with a high degree of glandular cells.

Heselmeyer-Haddad *et al* (2003) showed in a series of Pap smears of cervical precursor lesions of squamous cell origin that analysis of the *TERC* gene amplification is able to distinguish normal epithelium and low-grade dysplasia from the group of high-grade lesions with high specificity and sensitivity. In another paper, the same group showed that gain of *TERC* was found in 33% of cytological normal Pap smears from women who developed carcinoma *in situ* or invasive cervical squamous cancer in the future (Heselmeyer-Haddad *et al*, 2005). In the same paper, the authors were also able to show that all CIN1/CIN2 lesions with a diploid pattern for the *TERC* probe panel (CEP7–CEP3–*TERC*) eventually regressed to normal Pap smears, whereas the CIN1/CIN2 lesions with *TERC* amplification progressed. Lesions with a tetraploid pattern for the panel (four signals for each probe) could develop either way. The author concluded that the detection of gain and amplification of *TERC* in routinely collected Pap smears can assist in identifying lesions with a high progression risk. As the results of the current study indicate, *TERC* appears to play a similar important role in the carcinogenesis of cervical adenocarcinomas. Interestingly, even the patterns of gain found in the adenocarcinomas are very comparable to the patterns found in cervical dysplasias of squamous cell type (Heselmeyer-Haddad *et al*, 2003, 2005). The most common patterns observed for both of the cell types are low-level gains of *TERC* on a diploid background (3–5 signals of *TERC*). Given the similarity to the results of the studies of *TERC* in cervical squamous cell lesions, it seems to be a distinct possibility that analysis of *TERC* gain may also help to find women at risk for developing adenocarcinoma. An objective molecular marker in this patient group is of extreme importance because of the cytomorphologically difficult identification of these cells and the limited numbers of representative cells owing to technical problems in sampling these lesions. As shown in our study, many women with cervical adenocarcinoma presented with morphologically normal Pap smears, often only 1–2 years before diagnosis. A marker like *TERC* could have considerable impact on the detection of cervical adenocarcinomas at an earlier stage. Fluorescence *in situ* hybridisation analyses of precursor lesions, that is, adenocarcinomas *in situ* and dysplasias will be the next step to verify the potential of *TERC* as a marker of early detection and progression in cervical adenocarcinomas. We are therefore planning to conduct both retrospective studies with archival Pap smears and prospective randomised studies with routinely collected Pap smears.

## Figures and Tables

**Table 1:** Clinical details and HPV analysis of the 12 adenocarcinomas in the study

								**Follow-up**	
**Case no.**	**Cancer diagnosis**	**Last Pap smear year/ result**	**Age at diagnosis (years)**	**Tumour stage**	**Tumour differentiation**	**Lymph node involvement**	**HPV infection**	**Time (years)**	**Outcome**
1	1992	1990/normal	30	1B	Poor	No	HPV 16	13	AwoD
2	1992	1989/normal	46	1B	Poor	No	HPV 18	13	AwoD
3	1995	1992/normal	37	1B	Well	No	HPV 18	10	AwoD
4	1997	1987/normal	39	1B	Well	No	Not detected	8	AwoD
5	1992	1978/normal	50	2B	Well	Yes	Not detected	13	AwoD
6	1994	1992/normal	32	1B	Poor	No	HPV 18	11	AwoD
7	2000	1997/normal	62	1B	Well	No	HPV 45	4	DoD
8	2000	1998/normal	64	1B	Poor	No	Not detected	5	AwoD
9	1999	1996/normal	63	2B	Moderate	Yes	Not detected	6	AwoD
10	2000	1999/inflammation	53	1A	Well	No	HPV 16	5	AwoD
11	1997	1996/normal	45	1B	Well	No	HPV 18	7	DoD
12	1993	1992/normal	49	1A	Well	No	HPV 18	11	AwoD

AwoD=alive without disease; DoD=dead of disease; HPV=human papillomavirus.

**Table 2:** Results from interphase FISH analysis of the 12 cervical adenocarcinomas

							**Signals per cell: mean (range)**			**Relative *TERC vs***		
**Case no**	**Nuclei counted**	**‘Diploid’ (2-2-2) (%)**	**‘Nondiploid’^a^ (%)**	**‘Tetraploid’ (4-4-4) (%)**	**Nuclei with >2 *TERC* (%)**	**‘Nondiploid’ with >2 *TERC* (%)**	**CEP7**	**CEP3**	**TERC**	**CEP7**	**CEP3**	**Most common ‘nondiploid’ patterns**
1	376	33	67	1	66	99	2.2 (2–5)	2.2 (2–5)	3.0 (2–8)	1.4	1.4	(2-2-3) (2-2-4)
2	391	31	69	0	64	92	2.1 (2–4)	2.1 (2–4)	3.2 (2–7)	1.5	1.6	(2-2-4) (2-2-3) (2-2-5)
3	215	27	73	12	68	94	3.0 (2–6)	3.1 (2–8)	3.3 (2–9)	1.1	1.1	(4-4-4) (4-3-4) (4-3-3) (3-3-3) (3-4-4)
4	466	40	60	0	59	99	2.0 (1–4)	3.2 (2–5)	4.4 (2–12)	2.1	1.4	(2-4-6) (2-3-5) (2-4-5) (2-4-7)
5	336	68	32	1	27	86	2.1 (2–4)	2.1 (2–4)	2.4 (2–6)	1.1	1.1	(2-2-3) (2-2-4) (2-3-2)
6	641	69	31	1	28	92	2.0 (0–4)	2.3 (2–8)	2.6 (2–8)	1.3	1.1	(2-3-4) (2-3-5) (2-2-3) (2-3-3)
7	412	28	72	0	71	99	2.1 (2–4)	2.1 (2–8)	4.2 (2–15)	2.0	2.0	(2-2-5) (2-2-4) (2-2-6) (2-2-3)
8	626	46	54	1	54	98	2.0 (2–4)	2.2 (2–5)	2.9 (2–12)	1.4	1.3	(2-2-4) (2-2-3) (2-3-3) (2-2-5)
9	253	29	71	0	64	91	2.6 (2–5)	3.0 (2–7)	3.4 (2–11)	1.3	1.1	(2-2-3) (2-3-3) (2-2-4) (3-2-2) (3-4-4)
10	286	30	70	0	66	95	2.7 (2–7)	2.9 (2–7)	5.2 (2–22)	2.0	1.8	(2-2-4) (2-2-3) (2-2-5) (2-2-6)
11	393	79	21	4	18	83	2.2 (2–6)	2.2 (2–6)	2.3 (2–6)	1.04	1.04	(2-2-3) (4-4-4) (2-3-2)
12	208	20	80	0	79	99	1.9 (0–4)	2.0 (0–5)	3.0 (2–7)	1.6	1.5	(2-2-3) (2-2-4)

a Background level of deviation from (2-2-2) in normal nuclei is less than 2% (Heselmeyer-Haddad *et al*, 2003).

FISH=fluorescence *in situ* hybridisation.
